# Cadmium stress triggers dopamine signaling in duckweed (*Lemna turionifera* 5511) revealed by a fluorescent biosensor

**DOI:** 10.3389/fpls.2026.1833157

**Published:** 2026-06-01

**Authors:** Bosen Hao, Ziyang Qu, Yunwen Yang, Wenqiao Wang, Yun Xing, Yujie Han, Lin Yang

**Affiliations:** 1 Tianjin Key Laboratory of Animal and Plant Resistance, College of Life Sciences, Tianjin Normal University, Tianjin, China; 2 Department of Anatomy and Physiology, Laboratory of Anesthesiology, Shanghai Jiao Tong University, Ministry of Education, Shanghai Jiao Tong University School of Medicine, Shanghai, China; 3 State Key Laboratory of Forage Breeding-by-Design and Utilization, Institute of Botany, Chinese Academy of Sciences, Beijing, China; 4 MOE Key Laboratory of Bioinformatics, Tsinghua-Peking Joint Center for Life Sciences, and School of Life Sciences, Tsinghua University, Beijing, China

**Keywords:** cadmium stress, dopamine signaling, duckweed, fluorescent biosensor, genetically encoded biosensors

## Abstract

**Introduction:**

Cadmium (Cd) contamination poses a serious threat to aquatic ecosystems and human health. Duckweed has been recognized as a promising plant for phytoremediation of heavy metal–polluted water, yet the signaling mechanisms underlying its response to Cd stress remain poorly understood. Dopamine (DA), a catecholamine known as a neurotransmitter in animals, has recently been identified as a potential regulatory molecule in plants, but its dynamic response under heavy metal stress has not been clearly characterized.

**Methods:**

In this study, dopamine signaling dynamics in duckweed (*Lemna turionifera* 5511) were investigated using the genetically encoded fluorescent dopamine sensor GRAB-DA2h. Stable transgenic duckweed lines expressing the sensor were generated via *Agrobacterium*-mediated transformation, enabling real-time visualization of intracellular dopamine changes.

**Results:**

With Cd treatment, GRAB-DA2h fluorescence in root tissues increased within 1 minute and remained elevated during the imaging period, indicating a rapid early signaling response. Following a 24-hour exposure to 50 mM CdCl2, the protoplasts isolated from fronds exhibited an increase in fluorescence intensity from 345.18 to 391.14, while those derived from roots showed an increase from 112.75 to 140.68.

**Discussion:**

These findings provide direct evidence of dopamine signaling dynamics in duckweed under cadmium stress and demonstrate the applicability of genetically encoded biosensors for studying plant stress signaling. This work offers new insights into plant responses to heavy metal stress and may contribute to improving phytoremediation strategies.

## 1 Introduction

Cadmium (Cd) is a highly toxic heavy metal pollutant primarily released into the environment through industrial activities and intensive agricultural practices (Genchi et al., 2020). Its entry into ecological cycles is driven by key anthropogenic sources, including mining and smelting, the long-term application of cadmium-containing phosphate fertilizers, electronic waste disposal, and industrial wastewater discharge (Cheng et al., 2019). Due to its high mobility and bioavailability, cadmium readily disperses through aquatic systems, transfers within soil-plant systems, and ultimately bioaccumulates in top predators of the food chain (Wang R et al., 2023), posing serious threats to ecosystem integrity and human health (Satarug et al., 2003). Consequently, phytoremediation technologies has emerged as a promising strategy for mitigating cadmium contamination. However, the effectiveness of phytoremediation is fundamentally limited by plant sensitivity to cadmium stress, which severely inhibits essential physiological processes such as growth and photosynthesis (Zhang et al., 2021). Therefore, deciphering the signaling mechanisms underlying plant stress responses and manipulating key genes in these pathways are crucial for enhancing cadmium tolerance and improving remediation efficacy.

Unlike animals, plants are sessile organisms and must rely on rapid and sophisticated signaling systems to perceive and adapt to environmental challenges. Several signaling molecules-including glutamate, glutamine, γ-aminobutyric acid (GABA), and calcium-have been well-established as central mediators of plant stress responses (Toyota et al., 2018). In animals, the catecholamine dopamine (DA) functions as a critical neurotransmitter, modulating synaptic excitation and various behaviors. Intriguingly, DA is also ubiquitously present in plants, where it is increasingly recognized as a regulator of growth, development, and abiotic stress tolerance (Liu et al., 2020). It can directly scavenge reactive oxygen species (ROS), modulate antioxidant systems, and influence ion transporter activity to maintain cellular homeostasis under stress (Ahammed et al., 2020; Gao et al., 2020). Our previous work revealed that cadmium stress significantly suppresses endogenous DA biosynthesis in duckweed, suggesting a correlation between reduced DA levels and increased cadmium sensitivity (Wang W et al., 2023). Despite these findings, current understanding is largely based on endpoint measurements and exogenous application studies. The real-time, *in vivo* spatiotemporal dynamics of DA as a signaling molecule during stress remain largely unexplored in plants, representing a significant knowledge gap.

Technological advances in animals have enabled the real-time monitoring of DA dynamics through genetically encoded, G-protein–coupled receptor (GPCR)–based DA sensors, such as the dLight and GRABDA families, which allow high-resolution visualization of dopamine fluctuations (Patriarchi et al., 2018; Sun et al., 2020). These sensors overcome the spatiotemporal and specificity limitations of traditional methods by harnessing ligand-induced conformational changes in DA receptors to generate optical readout. This innovative raises a compelling and unresolved question: can similar high-resolution technologies be adapted for use in plants to monitor live DA signaling dynamics under stress? Addressing this gap requires a genetically tractable model system. Duckweed, with its small size, rapid proliferation, ease of genetic transformation, and established role in phytoremediation research, presents an ideal platform (Ekperusi et al., 2019). Moreover, the successful development of biosensor-expressing duckweed lines has demonstrated its suitability for real-time physiological monitoring (Ren et al., 2022), paving the way for the implementation of DA sensor technology in plant systems.

Here, we propose a novel research paradigm that integrates genetically encoded DA sensors with the duckweed model system (Ziegler et al., 2015). We hypothesize that cadmium exposure induces specific spatiotemporal patterns of DA signaling in duckweed, and that these dynamics changes are integral to the plant’s tolerance and cadmium accumulation capacity. To test this hypothesis, we generated transgenic *Lemna* lines expressing the DA sensor GRAB-DA2h, enabling real-time visualization of DA signaling dynamics during cadmium stress—a first in plant biology. We employed flow cytometry to identify putative “source and sink” tissues for DA metabolism and performed transcriptomic profiling to map genome-wide responses of DA metabolism and signaling pathways to cadmium challenge. This study revealed the real-time signaling role of DA in plant heavy metal stress responses, providing a theoretical and technical framework for developing signal-modulated, “smart” phytoremediation strategies.

## 2 Materials and methods

### 2.1 Duckweed cultivation

The duckweed (*Lemna turionifera* 5511) used in this study was originally collected from the Fengchan River in Tianjin, China, and cultured under the following conditions: a 16/8 h light/dark photoperiod with a light intensity of 95 µmol·m^-2^·s^-^¹, and a temperature cycle of 24 °C (day)/22 °C (night) (Yang et al., 2020). For Cd treatment, 2 mM CdCl_2_ working solution was prepared from a 0.1 M stock. This relatively high concentration (2 mM) was selected for live imaging to ensure a rapid and synchronized stress induction within minutes, enabling visualization of early DA dynamics at cellular resolution. For protoplast isolation and RNA-seq, a lower concentration (50 µM) was used to allow chronic stress adaptation over 24 h without triggering acute cytotoxicity, consistent with our previous physiological studies. All experiments included three independent biological replicates.

### 2.2 Plasmid construction and genetic transformation of duckweed

**Figure 1:**
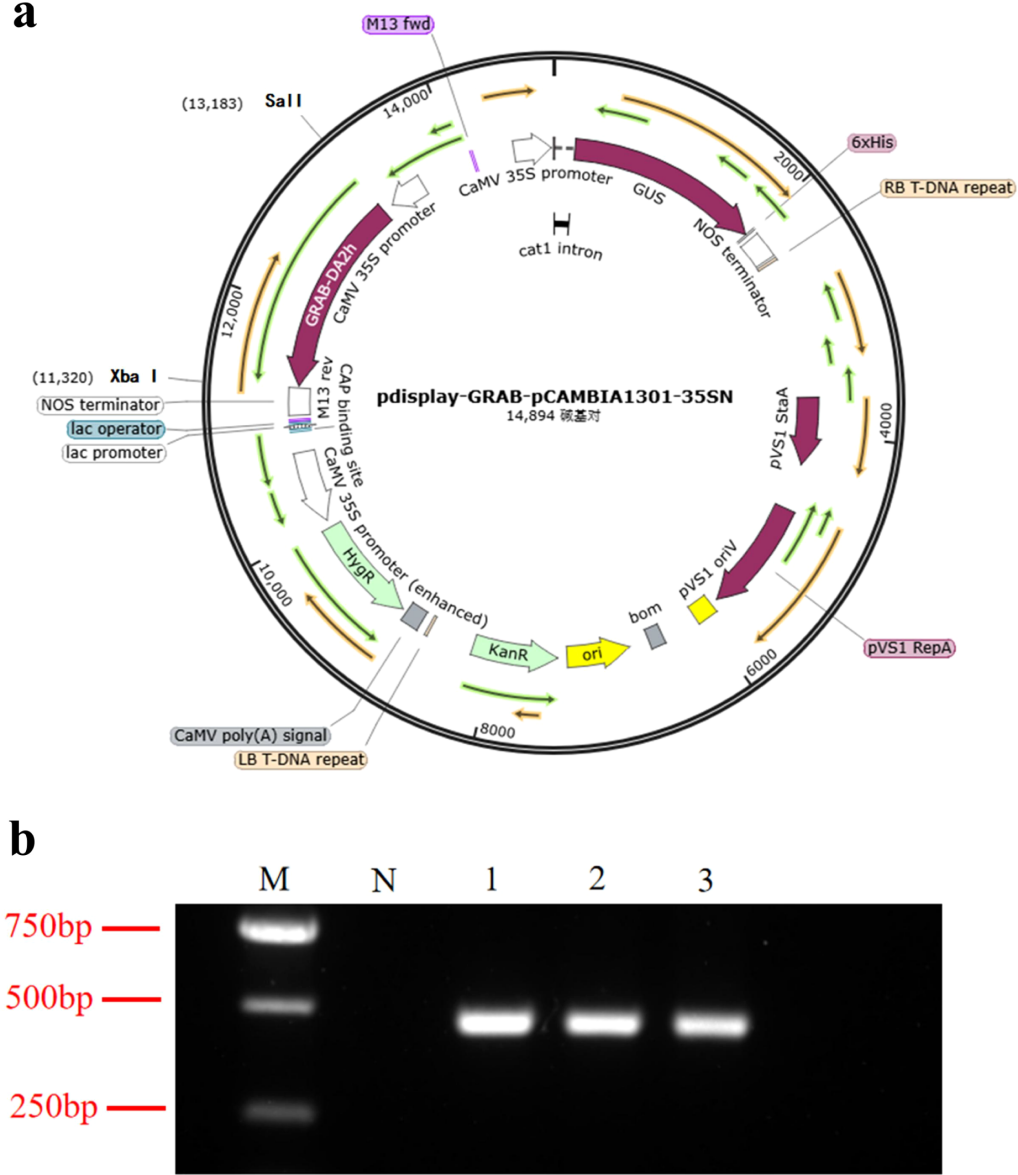
**(A)** Schematic representation of the recombinant vector construction. **(B)** PCR analysis: (1) Verification of the recombinant plasmid in *Agrobacterium tumefaciens*, using bacterial culture as a template. (2) Confirmation of GRAB-DA2h integration into the duckweed genome, using genomic DNA as a template. Lanes: M, DNA ladder; WT, wild-type duckweed; N, negative control (water); 1-3, independent transgenic lines (DA2h 1–3).

To visualize DA dynamics in living plant cells under Cd stress, we utilized the genetically encoded, G-protein-coupled receptor (GPCR)-based DA sensor GRAB-DA2h (Sun et al., 2020). For stable expression in duckweed (Lemna turionifera 5511), the GRAB-DA2h coding sequence was cloned into the binary vector pCAMBIA-1301 under the control of the constitutive CaMV 35S promoter (**Figure 1A**). The recombinant plasmid was introduced into Agrobacterium tumefaciens strain GV3101 via the freeze-thaw method (Havir and McHale, 1988) and subsequently used for duckweed transformation through an established Agrobacterium-mediated protocol (Yang et al., 2017).

For genetic transformation, duckweed calli were induced and co-cultivated with *A. tumefaciens* harboring the GRAB-DA2h construct, following an established protocol (Yang et al., 2017). Putative transgenic lines were selected on hygromycin-containing medium. Several independent transgenic lines were regenerated and propagated for subsequent analysis.

### 2.3 PCR analysis

Genomic DNA was isolated from GRAB-DA2h transgenic duckweed using a Plant Genomic DNA Extraction Kit (TIANGEN, Beijing, China). Polymerase chain reaction (PCR) was performed in a 20 μL reaction mixture containing 10 μL of Premix Taq, 2 μL of template DNA, 1 μL of forward primer, 1 μL of reverse primer, and 6 μL of ddH_2_O. The thermal cycling conditions were as follows: initial denaturation at 94 °C for 5 min; 30 cycles of denaturation at 94 °C for 1 min, annealing at 60 °C for 45 s, and extension at 72 °C for 1 min 40 s; followed by a final extension at 72 °C for 10 min. PCR products were analyzed by agarose gel electrophoresis. The primers used were:

F: ATGGAGACAGACACACTCCT

R: CTTGTACAGCTCGTCCATG

### 2.4 Laser scanning confocal microscopy of GRAB-DA2h transgenic duckweed

To monitor the real-time dynamics of DA signals in duckweed roots under cadmium stress, laser scanning confocal microscopy (N-storm, Nikon) was employed. The GRAB-DA2h-GFP was excited at 488 nm, and emission was collected at 500–550 nm. To validate sensor specificity, parallel experiments were performed using transgenic duckweed pretreated with 1 mg/L haloperidol (a D2-like dopamine receptor antagonist) for 2 h prior to CdCl_2_ exposure, followed by identical imaging acquisition (**Supplementary Figure 1**). Images were captured at 1-minute intervals to record the dynamic changes in fluorescence signals. Fluorescence intensity analysis was performed using ImageJ software (Fiji).

### 2.5 Protoplast isolation and flow cytometry analysis

Roots and fronds were collected separately after treated with or without 50 μM CdCl_2_ for 24 h. Protoplast isolation and Leadmium™ Green AM staining were performed as described as Ma et al., 2024. All experiments were carried out with three independent biological replicates. Roots were fixed in 95% ethanol for 15 min and rinsed. Protoplasts were isolated by incubating root tissues in an enzyme solution (1% cellulose and 1% pectinase in 0.4 M mannitol, pH 5.7) at 37 °C in the dark for 60 min. The protoplast suspension was filtered through a 400-mesh cell strainer and washed three times with DPBS. For cadmium quantification, protoplasts were stained with the cadmium-sensitive fluorescent dye Leadmium™ Green AM (Invitrogen) according to the manufacturer’s instructions. Fluorescence intensity of at least 5,000 protoplasts per sample was analyzed using an imaging flow cytometer (FlowSight^®^; Merck Millipore, Darmstadt, Germany). Data were processed using IDEAS^®^ software.

### 2.6 RNA sequencing and analysis

Duckweed plants were pretreated with 100 µM exogenous dopamine and subsequently treated with or without 50 µM CdCl_2_ for 24 h, three independent biological replicates were established. Total RNA was extracted using the RNeasy Plant Mini Kit (QIAGEN). All subsequent procedures, library preparation, sequencing, and bioinformatic analysis, were conducted by Novogene Bioinformatics Technology Co., Ltd. (Chaoyang, Beijing, China). Total RNA was isolated using the QIAGEN Plant Total RNA Extraction Kit (QIAGEN, Beijing, China), and its integrity was assessed with an Agilent 2100 Bioanalyzer (Agilent Technologies, Santa Clara, CA, USA). Qualified RNA samples were used to construct sequencing libraries, which were then subjected to paired-end sequencing on an Illumina NovaSeq 6000 platform (Illumina, San Diego, CA, USA).

The raw sequencing reads were processed through a standard bioinformatics pipeline, which included quality control checks, trimming of low-quality sequences, and alignment of the clean reads to the reference genome. Differential gene expression analysis was performed using the DESeq2 software by comparing the read counts of genes across samples to identify significantly up- or down-regulated genes. To interpret the biological implications, functional annotation and enrichment analyses were carried out using tools such as Gene Ontology (GO), the Kyoto Encyclopedia of Genes and Genomes (KEGG), and pathway analysis. Gene functional annotation was based on the GO database, the KEGG Orthology (KO) database, and the manually annotated and reviewed protein sequence database (Swiss−Prot). Gene expression levels were quantified.

### 2.7 Statistical analysis

All experiments were performed with at least three independent biological replicates. Data analysis was conducted using Microsoft Excel 2021. Data are presented as mean ± standard error (SE). Statistical analysis was performed using GraphPad Prism 9.0. The significance of differences between two groups was assessed by Student’s t-test (two-tailed). Multiple comparisons were analyzed by one-way or two-way ANOVA followed by Tukey’s *post hoc* test (T-test). with significance levels indicated by asterisks (*p < 0.05, **p < 0.01, ***p < 0.001, ****p < 0.0001, ns: not significant).

## 3 Results

### 3.1 Generation and molecular identification of GRAB-DA2h transgenic duckweed

As schematically illustrated in **Figure 1A**, the GRAB-DA2h, encoding G-protein-coupled receptor (GPCR)-based DA sensor, was cloned into a binary vector under the control of the constitutive *CaMV 35S* promoter, ensuring strong and ubiquitous expression.

Molecular confirmation of transgene integration was performed by PCR on genomic DNA extracted from putative transgenic lines. As shown in **Figure 1B**, the expected amplicon was successfully detected in three independent transgenic lines (DA2h 1–3), but not in wild-type (WT) duckweed or negative controls. This confirms the successful generation of stable transgenic duckweed lines expressing the GRAB-DA2h sensor, providing a foundational tool for real-time DA monitoring.

### 3.2 Real-time dynamics of dopamine fluorescence in response to cadmium stress

**Figure 2:**
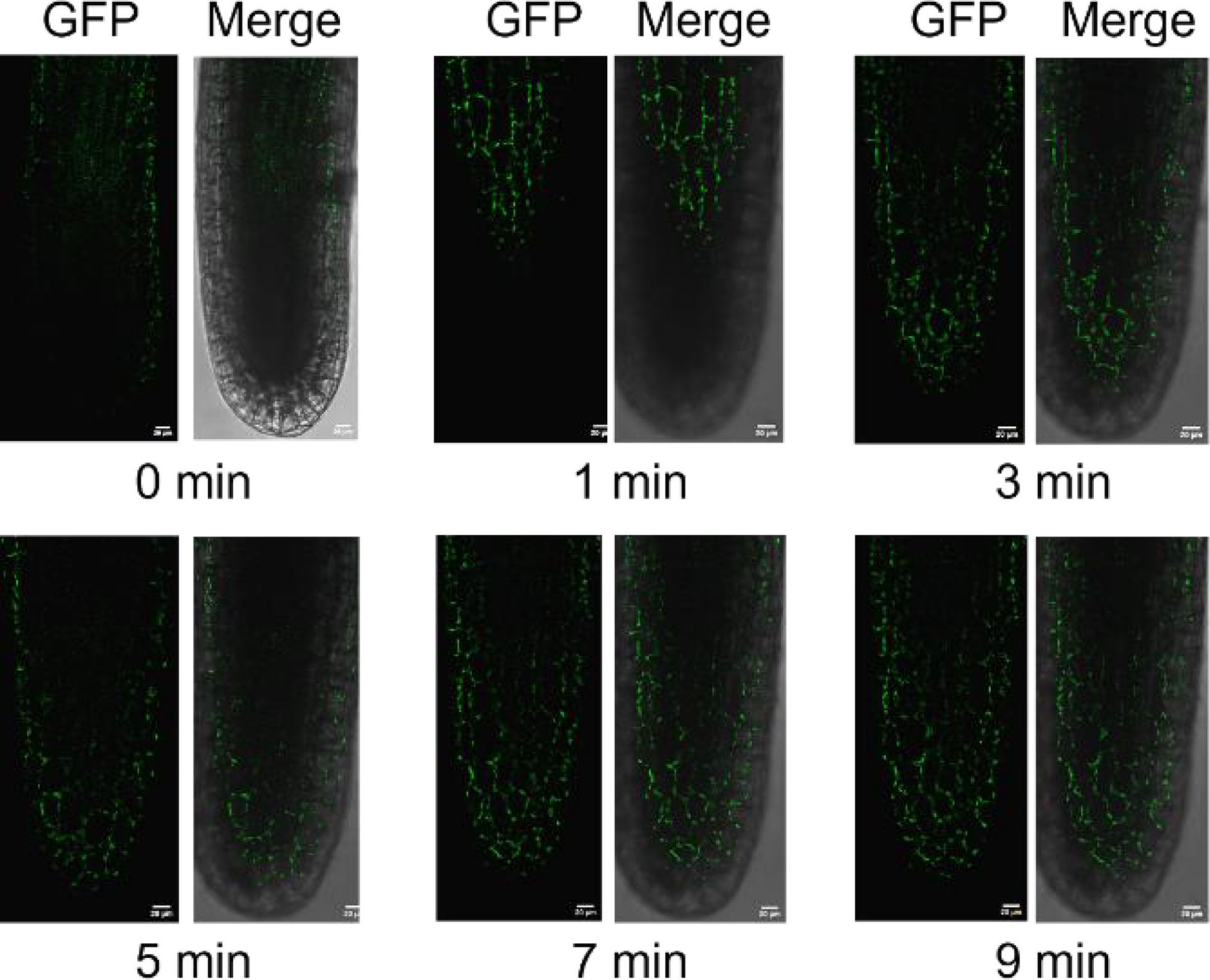
Sequential images show spatiotemporal changes in dopamine-associated fluorescence in transgenic duckweed roots expressing the dopamine sensor at indicated time points after treatment.

To capture the spatiotemporal dynamics of DA signaling upon Cd exposure, we performed live confocal microscopy on roots of 6-day-old GRAB-DA2h transgenic duckweed. Following treatment with 2 mM CdCl_2_, time-lapse imaging was conducted at 1-min intervals. As shown in **Figure 2**, a rapid and sustained increase in GRAB-DA2h fluorescence intensity was observed in root tissues, beginning within 1 min post-treatment and intensifying over the 9-min observation period. As shown in **Figure 2**, a notable increase in fluorescence intensity was observed in the root tissues of GRAB-DA2h transgenic duckweed following CdCl_2_ treatment. This enhancement in the fluorescence signal intensified over the duration of the experiment, demonstrating a time-dependent response.

To validate the specificity of the GRAB-DA2h dopamine fluorescent sensor and exclude non-specific fluorescence interference caused by direct cadmium ion action or other factors, a control experiment with haloperidol pretreatment was performed. As shown in **Supplementary Figure 1**, the GFP fluorescence signal in the roots of haloperidol- pretreated duckweed remained at a low level throughout the same cadmium stress treatment, without obvious time-dependent enhancement.

This observation directly indicates that the GRAB-DA2h transgenic duckweed exhibits a significant increase in DA fluorescence signals in response to cadmium stress, confirming the sensor’s capability to report dynamic changes in DA levels *in vivo*.

### 3.3 Quantitative analysis of dopamine content in protoplasts under cadmium stress

**Figure 3:**
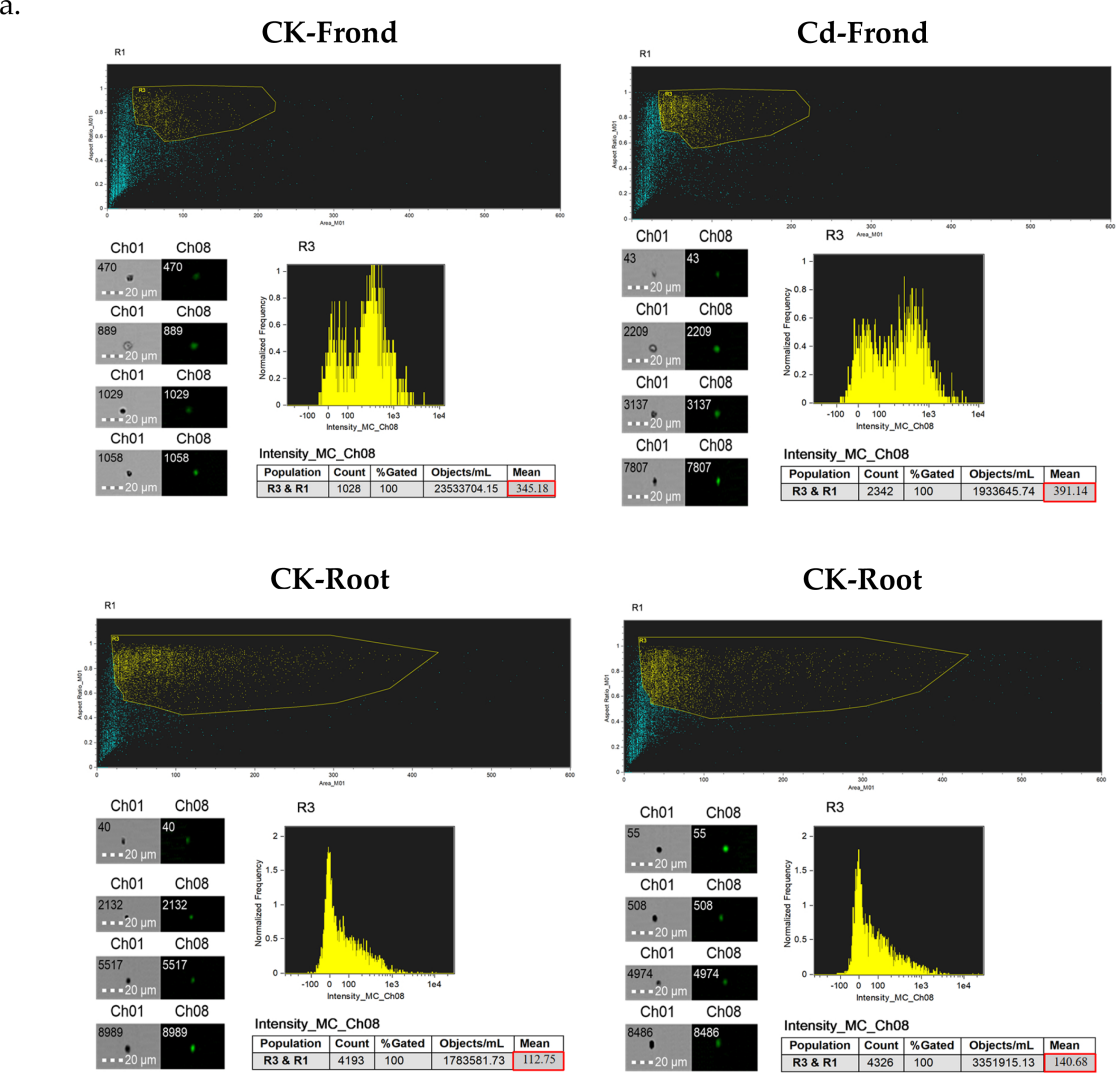
Representative bright-field (Ch01) and fluorescence (Ch08, 488 nm excitation) images and quantitative mean fluorescence intensity of protoplasts. Protoplasts were isolated from both roots and fronds of transgenic GRAB-DA2h duckweed following 24-hour exposure to 50 μM CdCl_2_ (scale bar = 20 µm). Results show enhanced dopamine accumulation in Cd-treated protoplasts compared to controls. CK: control; Cd: cadmium-treated.

To quantitatively assess the cellular-level changes in DA levels under cadmium stress, the DA fluorescence intensity in the protoplasts from DA duckweed was analyzed using the FlowSight imaging flow cytometer with an excitation wavelength of 488 nm. As shown in **Figure 3**, Cd-treated protoplasts exhibited significantly higher mean fluorescence intensity compared to controls in both tissue types. In frond-derived protoplasts, the mean intensity increased from 345.18 (control) to 391.14 (Cd-treated); in protoplasts of root, it increased from 112.75 to 140.68 (p < 0.05, t-test). These data corroborate the confocal imaging results and demonstrate that cadmium induces a systemic up-regulation of cellular DA levels, reinforcing the role of DA as a widespread signaling molecule under heavy metal stress. These data corroborate the confocal imaging results and demonstrate that cadmium induces a systemic up-regulation of cellular DA levels, reinforcing the role of DA as a widespread signaling molecule under heavy metal stress.

### 3.4 Dopamine extracellular vesicles (EVs) secretion under cadmium stress

**Figure 4:**
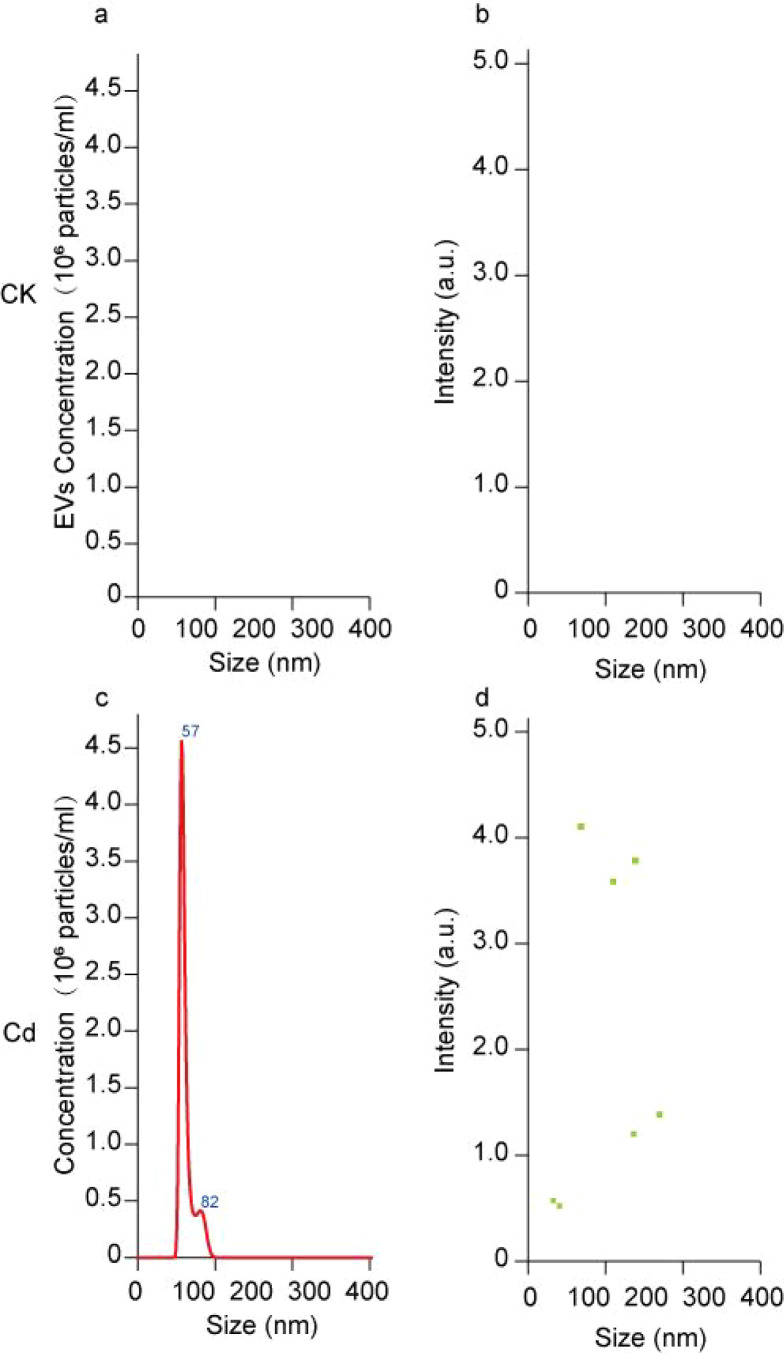
The size and quantity of EVs from GRAB-DA2h were determined using NTA. **(A)** size distribution histogram for control medium; **(B)** fluorescence intensity histogram (488 nm) for control; **(C)** size distribution histogram for Cd−treated medium; **(D)** fluorescence intensity histogram (488 nm) for Cd−treated.

To investigate the extracellular secretion of dopamine (DA) in response to cadmium stress, we employed nanoparticle tracking analysis (NTA) to monitor the release of extracellular vesicles (EVs) from GRAB-DA2h transgenic duckweed following cadmium treatment. As shown in **Figures 4A, B**, in the absence of cadmium stress, no significant EV peaks or fluorescence signals were detected in the supernatant of the culture medium. However, upon 24-hour exposure to 50 μM CdCl_2_, a distinct EV population emerged with a size distribution predominantly within the 50-100 nm range. Concurrently, fluorescence intensity analysis at 488 nm revealed a significant increase in signal intensity, indicating the presence of dopamine-containing EVs (**Figures 4C, D**). Notably, the fluorescent EVs were predominantly concentrated within the 50-100 nm size range, demonstrating that cadmium stress specifically induces the secretion of DA-loaded EVs. This finding provides evidence that cadmium stress triggers the extracellular release of dopamine via EVs.

### 3.5 Cadmium stress and exogenous dopamine co-modulate dopamine metabolism and its mediated signaling network in duckweed

**Figure 5:**
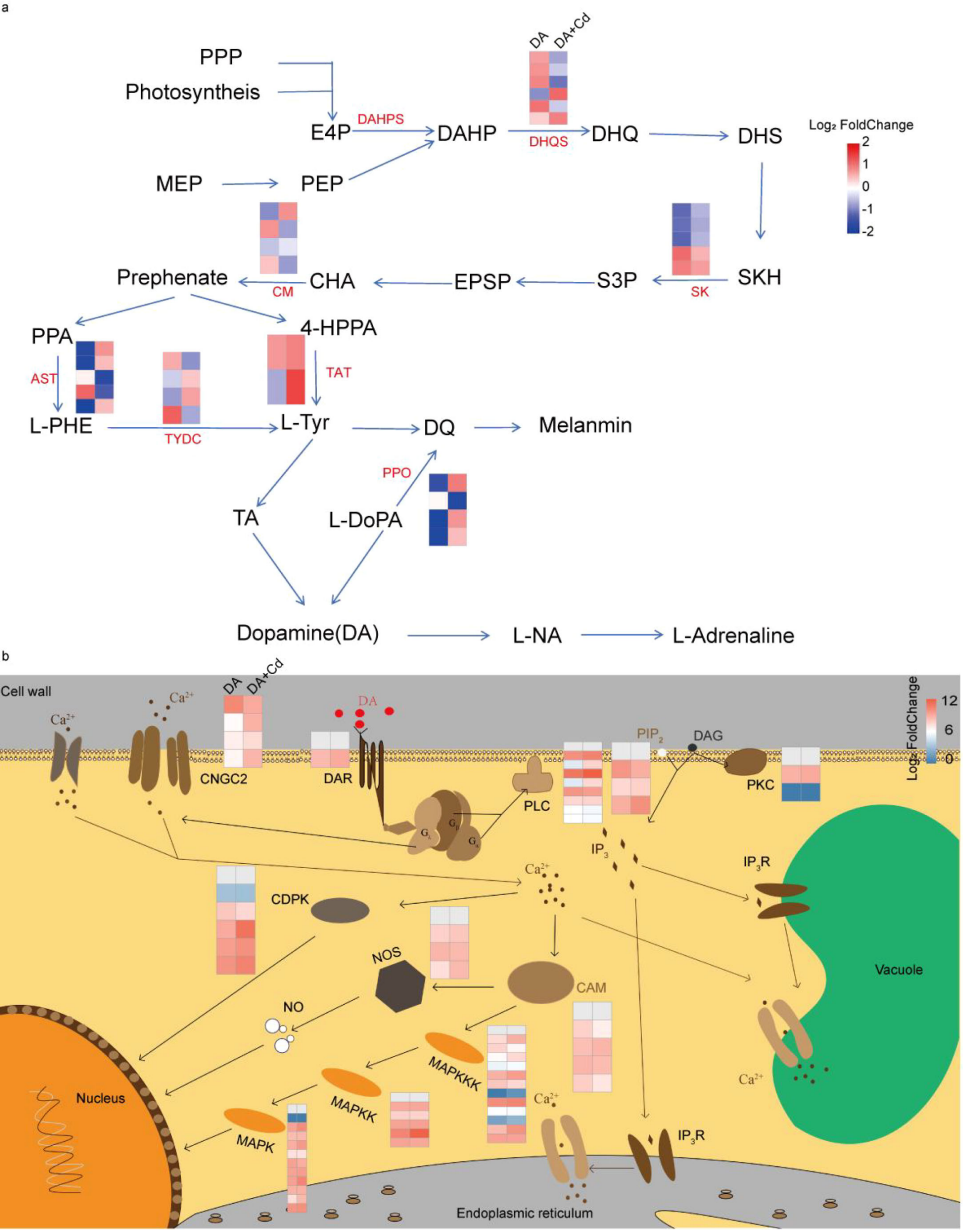
Dopamine metabolism and signaling in duckweed under cadmium stress and exogenous dopamine treatment. **(A)** Metabolic pathway map under DA and DA+CdCl_2_. Key catalytic enzymes are labeled in red. The pathway outlines precursor supply (PPP and MEP pathways), aromatic amino acid biosynthesis, dopamine synthesis, and downstream branches. pentose phosphate pathway (PPP); Photosynthesis (photosynthetic pathway); erythrose-4-phosphate (E4P); 3-deoxy-D-arabino-heptulosonate-7-phosphate synthase (DAHPS); 3-deoxy-D-arabino-heptulosonate-7-phosphate (DAHP); 3-dehydroquinate synthase (DHQS); 3-dehydroquinate (DHQ); 3-dehydroshikimate (DHS); methylerythritol phosphate pathway (MEP); phosphoenolpyruvate (PEP); Prephenate (prephenic acid); chorismate (CHA); chorismate mutase (CM); 5-enolpyruvylshikimate-3-phosphate (EPSP); shikimate-3-phosphate (S3P); shikimate kinase (SK); shikimate (SKH); phenylpyruvate (PPA); aromatic amino acid transaminase (AST); L-phenylalanine (L-PHE); tyrosine decarboxylase (TYDC); 4-hydroxyphenylpyruvate (4-HPPA); tyrosine aminotransferase (TAT); L-tyrosine (L-Tyr); dopaquinone (DQ); melanin (Melanmin); polyphenol oxidase (PPO); L-3,4-dihydroxyphenylalanine (L-DoPA); tyramine (TA); dopamine (DA); L-norepinephrine (L-NA); L-adrenaline (L-adrenaline). This pathway covers precursor supply, aromatic amino acid biosynthesis, dopamine synthesis, and downstream metabolic branches. The color bar represents log2 fold change, with red indicating up-regulated expression and blue indicating down-regulated expression. **(B)** Signaling pathway map under DA and DA+CdCl_2_ treatment. The diagram illustrates signal transduction from plasma membrane receptors and channels through intracellular cascades (Ca²^+^ signaling, PLC-IP_3_, NO synthesis, MAPK) to nuclear regulation. ACA8, autoinhibited Ca²^+^-ATPase 8; CaM, calmodulin; CNGC2, cyclic nucleotide-gated channel 2; DAR, dopamine receptor; IP_3_R, inositol 1,4,5-trisphosphate receptor; NOS, nitric oxide synthase; PKC, protein kinase **(C)** The color bar represents log2 fold change, and color intensity indicates the magnitude of expression change.

To elucidate the metabolic and signaling adjustments underlying the observed DA dynamics, exogenous DA was applied with or without Cd for 24 h. Transcriptomic analysis on duckweed has been studied. As summarized in **Figure 5A**, Cd stress triggered a transcriptional change in the DA biosynthetic pathway. Within the upstream shikimate pathway, key genes were differentially regulated: the expression level of shikimate kinase (SK) was down-regulated to 0.73-fold of the control, while 3-dehydroquinate synthase (DHQS) was down-regulated to 0.86-fold of the control. This selective modulation likely adjusts the metabolic flux toward aromatic amino acid synthesis. Accordingly, downstream genes involved in aromatic amino acid metabolism were notably activated: tyrosine decarboxylase (TYDC) and polyphenol oxidase (PPO), two enzymes critical for converting L-tyrosine to dopamine, were strongly up-regulated to 2.25-fold and 2.04-fold of the control, respectively. This pattern is consistent with an enhanced precursor flux driving DA biosynthesis under Cd challenge.

DA signaling network was engaged described as **Figure 5B**. The expression of a putative DA receptor (DAR) homolog was up-regulated to 0.51-fold of the control, indicating enhanced perception of the DA signal at the plasma membrane. Downstream calcium signaling components were modulated: phospholipase C (PLC) transcript level was 0.75-fold of the control, suggesting a potential alteration of IP_3_-mediated Ca²^+^ release, while the cyclic nucleotide-gated channel 2 (CNGC2) was downregulated to 0.14-fold of the control. Key intracellular transducers showed divergent changes—calcium-dependent protein kinase (CDPK) was slightly increased to 1.08-fold of the control, whereas calmodulin (CaM) declined to 0.24-fold. This transcriptional response was integrated with the modulation of the MAPK cascade, whose components (MAPKKK, MAPKK, and MAPK) exhibited expression levels ranging from 0.52- to 0.90-fold of the control, and with an alteration in nitric oxide synthase (NOS) expression (0.39-fold of the control). Collectively, this multi-layered transcriptional response highlights how DA signaling interfaces with canonical Ca²^+^, MAPK, and NO pathways to coordinate an adaptive response to cadmium stress.

These changes demonstrate that DA+Cd treatment enhances dopamine-receptor perception and cooperatively activates Ca²^+^ signaling, NO signaling, and the MAPK cascade, constructing a multi-layered signaling network that mediates the adaptive response of duckweed to cadmium stress.

## 4 Discussion

In animal systems, dopamine (DA) is a critical neurotransmitter that modulates a wide range of physiological and behavioral processes through finely regulated spatiotemporal signaling (Liu et al., 2020). The recent development of genetically encoded GPCR-based DA sensors, such as dLight and GRAB-DA families, has enabled real-time, high-resolution visualization of DA dynamics *in vivo*, revealing its distribution and functional roles within neural circuits (Patriarchi et al., 2018; Sun et al., 2020). In contrast, the study of DA as a signaling molecule in plants has been hindered by the lack of tools for live monitoring. In this study, we report the first successful implementation of a genetically encoded DA sensor (GRAB-DA2h) in plants, using duckweed as a model. Our real-time imaging revealed that cadmium stress triggers a rapid and pronounced DA signal in root tissues, initiating from the root tip and propagating spatially over time. This finding establishes DA as a dynamic signaling agent in plant stress responses and opens a new avenue for studying catecholamine signaling in plants with unprecedented spatiotemporal precision (Fichman and Mittler, 2021).

### 4.1 Methodological considerations: limitations and specificity of the GRAB-DA2h sensor in plants

Although genetically encoded fluorescent sensors represent a revolutionary breakthrough for plant signaling research, the application of animal-derived GPCR-based sensors in plant cells entails inherent limitations that warrant careful consideration (Sadoine et al., 2021). First, the efficiency of expression, folding, and membrane targeting of animal GPCRs may be lower in plant cells than in their native hosts, and plant-specific post-translational modifications may affect receptor conformation and ligand-binding affinity. Second, the intracellular environment of plant cells differs markedly from that of animal cells in pH, ionic strength, and redox state, all of which may alter the fluorescence response characteristics and kinetic parameters of the sensor. Third, although the GRAB-DA2h sensor used here is a conformation-based single-molecule probe that does not rely on downstream signal transduction, non-specific interactions between the sensor and endogenous plant proteins cannot be entirely excluded. Finally, long-term expression of the sensor may exert potential perturbations on normal plant physiology; although no growth phenotypic abnormalities were observed in our transgenic duckweed lines, this aspect requires further assessment (Sadoine et al., 2021).

In light of these limitations, the specificity of the GRAB-DA2h sensor in the complex metabolic environment of plants is of paramount importance. GRAB-DA2h was engineered from the human dopamine D2 receptor, and its affinity and specificity for DA have been well validated in animal systems (Sun et al., 2020). However, plants contain other catecholamines such as norepinephrine and epinephrine, as well as abundant phenolic compounds and secondary metabolites. Although the concentrations of these compounds are typically much lower than that of DA, they may rise significantly under certain stress conditions and potentially interact with the sensor, leading to false-positive signals. For instance, high concentrations of L-DOPA (the precursor of DA) and tyramine may weakly interact with GRAB-DA2h, albeit with affinities several orders of magnitude lower than that of DA. To address this concern, we performed haloperidol-pretreatment control experiments, which confirmed that the cadmium-induced fluorescence enhancement was specifically mediated by endogenous DA signaling. This pharmacological validation minimized the contribution of non-specific signals and ensured the reliability of our results. Together, these considerations indicate that while technical caveats exist, they do not undermine the core conclusions of this study.

### 4.2 Integration of rapid DA signaling dynamics with long-term transcriptional reprogramming

A notable feature of this study is the acquisition of experimental results at two distinct time scales: live imaging revealed rapid, minute-scale DA signal responses, whereas transcriptomic profiling captured gene expression changes after 24 h of cadmium stress. These two temporal dimensions are intrinsically linked and serve distinct but complementary functions in duckweed Cd stress responses (Johns et al., 2021). The rapid DA signal likely represents an early sensing and warning system that is activated within minutes of Cd entry into cells, triggering immediate protective responses such as activation of antioxidant systems, modulation of ion transporter activity, and maintenance of cellular redox homeostasis (Akula and Mukherjee, 2020; Wang W et al., 2023). The 24 h transcriptomic changes, in turn, reflect long-term adaptive mechanisms involving genome-wide transcriptional reprogramming, including metabolic pathway adjustments, cell wall modification, and enhanced heavy metal chelation and detoxification. We propose that the rapid DA signal may function as an upstream second messenger that initiates and integrates downstream signaling cascades, ultimately leading to long-term transcriptional changes. This cascade, progressing from rapid signal transduction to sustained gene expression, would allow plants to temporally and spatially coordinate their stress responses, enabling a seamless transition from acute defense to long-term adaptation (Zhang et al., 2023). This hypothesis remains to be tested in future work.

### 4.3 Dopamine metabolism and signaling network under cadmium stress

Transcriptomic analysis provided mechanistic insights into the metabolic and signaling adjustments underlying the observed DA dynamics. Key enzymes in the DA biosynthetic pathway, including tyrosine decarboxylase (TYDC) and polyphenol oxidase (PPO), were significantly up-regulated, indicating that Cd stress enhances the metabolic flux toward DA production (**Figure 5A**). Concurrently, 3-dehydroquinate synthase (DHQS), the first committed enzyme of the shikimate pathway, was down-regulated to 0.86-fold of the control level. This down-regulation may represent a metabolic reprogramming strategy: by reducing carbon flux through the shikimate pathway toward lignin, flavonoids, and other aromatic secondary metabolites, duckweed may channel more precursors (e.g., phosphoenolpyruvate and erythrose-4-phosphate) toward tyrosine and DA biosynthesis, thereby meeting the increased demand for DA as a signaling molecule under stress conditions.

In addition, under the combined DA and Cd treatment (**Figure 5B**), a putative dopamine receptor (DAR) homolog was up-regulated, along with downstream components including calcium-dependent protein kinases (CDPKs) and phospholipase C (PLC), suggesting that DA signaling may interface with canonical Ca²^+^-mediated cascades. This is reminiscent of glutamate signaling in plants, where glutamate activates GLR channels to initiate systemic Ca²^+^ waves and defense responses (Toyota et al., 2018). Notably, cyclic nucleotide-gated channel 2 (CNGC2) was strongly down-regulated (0.14-fold) under DA+Cd treatment. As a non-selective cation channel that mediates Ca²^+^ and other cation influx, CNGC2 down-regulation may serve a protective function by limiting Cd²^+^ entry and reducing intracellular Cd accumulation, while simultaneously fine-tuning the amplitude and duration of Ca²^+^ signals to prevent excessive activation that could lead to programmed cell death. This pattern is consistent with a modulatory role of DA signaling in stress adaptation. The concurrent modulation of MAPK cascade components and nitric oxide synthase (NOS) further supports that DA signaling is integrated into a broader network of stress-responsive pathways (Liu et al., 2020; Akula and Mukherjee, 2020). We emphasize that the signaling network shown in **Figure 5B** was delineated under exogenous DA co-treatment; whether endogenous Cd-induced DA mobilizes an identical network requires further investigation.

### 4.4 Extracellular secretion of DA via vesicles and future perspectives

Our finding that Cd stress specifically induces the secretion of DA-containing extracellular vesicles (EVs) suggests a potential mechanism for intercellular DA signaling in plants. Although research on plant EVs remains in its early stages, parallels can be drawn from animal systems, where neurotransmitter loading into vesicles is mediated by vesicular monoamine transporters (VMATs) and EV cargo sorting relies on the ESCRT machinery and lipid raft-mediated pathways. Whether analogous mechanisms operate in plants to load DA into multivesicular bodies (MVBs) for subsequent secretion awaits future investigation.

Plant stress signaling typically involves the coordinated action of multiple pathways to ensure an effective and balanced response (Gonzales and Kelley, 2025). Our data indicate that DA signaling does not operate in isolation but interacts with Ca²^+^, MAPK, and NO signaling modules. Such multi-pathway integration may allow plants to fine-tune their responses to heavy metal stress, optimizing resource allocation between growth and defense. The capacity of DA to modulate this network positions it as a versatile regulator in plant environmental adaptation, with potential relevance to both abiotic and biotic stress tolerance (Cao et al., 2023; Carmo et al., 2025).

In conclusion, this study provides the first real-time visualization of DA signaling in plants and demonstrates its activation under cadmium stress. By integrating live imaging, single-cell quantification, and transcriptomics, we reveal that DA functions within a coordinated signaling network involving Ca²^+^, MAPK, and NO pathways. These findings advance our understanding of plant catecholamine signaling and highlight DA as a promising target for engineering stress-resilient crops. Future work should focus on identifying the precise molecular targets of DA in plants, elucidating its crosstalk with other signaling hormones, and exploring its potential in enhancing phytoremediation efficiency.

## Data Availability

The datasets presented in this study can be found in online repositories. The names of the repository/repositories and accession number(s) can be found in the article/**Supplementary Material**.
