# Supplementary material for: Cadmium stress triggers dopamine signaling in duckweed (Lemna turionifera 5511) revealed by a fluorescent biosensor
Source: Front Plant Sci. 2026 Jun 1;17:1833157. doi: 10.3389/fpls.2026.1833157 (PMC13265539; doi:10.3389/fpls.2026.1833157)
Supplement: Supplementary file 1 [file Table1.docx]

| Table S1 Changes in Gene Expression Related to the DA Signaling Network | | | | | |
| --- | --- | --- | --- | --- | --- |
| Gene_Id | Description | DA_  Readcount | DA+Cd_ Readcount | log2Fold Change | Pval |
| Cluster-4169.9780 | cyclic nucleotide gated channel | 2495.666667 | 1064.083 | -1.22904 | 0.029255 |
| Cluster-4169.6994 | cyclic nucleotide gated channel | 121.3333333 | 829.6667 | 2.763452 | 0.008795 |
| Cluster-4169.1849 | cyclic nucleotide gated channel | 166.03 | 291.32 | 0.807441 | 0.112887 |
| Cluster-4169.4344 | cyclic nucleotide gated channel | 132.3333333 | 789 | 2.566815 | 0.001945 |
| Cluster-4169.14074 | CDPK-related kinase 1 | 10.12666667 | 9.45 | -0.09052 | 0.898648 |
| Cluster-4169.9872 | CDPK-related kinase 5 | 496.6666667 | 320.5333 | -0.63021 | 0.02008 |
| Cluster-4169.8221 | CDPK-related kinase 3 | 1011 | 4937 | 2.286718 | 0.013888 |
| Cluster-4169.6859 | CDPK-related kinase 1 | 1549.026667 | 2173.867 | 0.488634 | 6.06E-05 |
| Cluster-4169.6406 | CDPK-related kinase 2 | 1914.333333 | 3120.79 | 0.704778 | 0.001183 |
| Cluster-4169.13920 | nitric oxide synthase-interacting protein | 387.6666667 | 491.6667 | 0.342078 | 0.000617 |
| Cluster-4169.5070 | nitric-oxide synthase | 800 | 683 | -0.22781 | 0.248404 |
| Cluster-4169.4363 | nitric-oxide synthase activity | 253.33 | 718.85 | 1.500995 | 0.000364 |
| Cluster-4169.13765 | Calmodulin-binding protein 60 C | 377.6666667 | 169.3333 | -1.15257 | 0.008089 |
| Cluster-4169.4425 | calcium ion binding | 580.3333333 | 796 | 0.455214 | 0.014439 |
| Cluster-4169.7541 | calcium-binding protein CML | 752.3333333 | 619 | -0.28102 | 0.706359 |
| Cluster-4169.4294 | Calmodulin-binding protein 60 C | 418.69 | 217.73 | -0.94017 | 0.088014 |
| Cluster-1422.0 | mitogen-activated protein kinase 15 | 0 | 0 | 0 | 0 |
| Cluster-4169.10627 | mitogen-activated protein kinase 3 | 1191.33 | 2330.06 | 0.967203 | 0.01547 |
| Cluster-4169.12625 | mitogen-activated protein kinase 1/2 | 637.6666667 | 723.3333 | 0.181591 | 0.152121 |
| Cluster-4169.9969 | mitogen-activated protein kinase 4 | 942.3333333 | 1696.943 | 0.847949 | 0.004434 |
| Cluster-4169.14392 | mitogen-activated protein kinase-binding protein 1 | 226 | 228 | 0.012655 | 0.949763 |
| Cluster-4169.11273 | mitogen-activated protein kinase 7 | 1308.553333 | 972.5867 | -0.42769 | 0.020234 |
| Cluster-4169.6365 | mitogen-activated protein kinase 6 | 1238.833333 | 1477.097 | 0.253594 | 0.022742 |
| Cluster-4169.8274 | mitogen-activated protein kinase 8 | 1352.21 | 2835.537 | 1.067745 | 0.003371 |
| Cluster-4169.9030 | mitogen-activated protein kinase 16 | 853.7566667 | 863.0167 | 0.015545 | 0.913566 |
| Cluster-4169.6478 | mitogen-activated protein kinase 4 | 259.9833333 | 707.99 | 1.441808 | 0.013071 |
| Cluster-4169.6356 | mitogen-activated protein kinase 8 | 1734.716667 | 2056.66 | 0.245473 | 0.186931 |
| Cluster-4169.11364 | mitogen-activated protein kinase kinase 1 | 990.6666667 | 1089.667 | 0.137283 | 0.438225 |
| Cluster-4169.3779 | mitogen-activated protein kinase kinase 3 | 357.8033333 | 334.4933 | -0.09691 | 0.456156 |
| Cluster-4169.10295 | mitogen-activated protein kinase kinase 4/5 | 1262.503333 | 1323 | 0.067474 | 0.557703 |
| Cluster-4169.8341 | mitogen-activated protein kinase kinase 2 | 2703.04 | 8269.797 | 1.61291 | 0.000104 |
| Cluster-4169.7499 | mitogen-activated protein kinase kinase 9 | 1193.666667 | 1085 | -0.13758 | 0.70613 |
| Cluster-4169.12974 | Mitogen-activated protein kinase kinase kinase 1 | 284.6666667 | 830.6667 | 1.541673 | 0.004434 |
| Cluster-4169.12176 | Mitogen-activated protein kinase kinase kinase NPK1 | 347.4966667 | 136 | -1.34697 | 0.006955 |
| Cluster-4169.13093 | Mitogen-activated protein kinase kinase kinase 17 | 121.6666667 | 299 | 1.290219 | 0.026435 |
| Cluster-4169.14026 | Mitogen-activated protein kinase kinase kinase A | 58.66666667 | 78 | 0.404927 | 0.400012 |
| Cluster-4169.10706 | Mitogen-activated protein kinase kinase kinase 1 | 869.3433333 | 1509.667 | 0.795529 | 0.000219 |
| Cluster-4169.2407 | Mitogen-activated protein kinase kinase kinase YODA | 492.3333333 | 314.3333 | -0.64569 | 0.015721 |
| Cluster-4169.4255 | mitogen-activated protein kinase kinase kinase 1 | 0 | 1.333333 | 1.222392 | 0.183503 |
| Cluster-4169.10560 | Mitogen-activated protein kinase kinase kinase 5 | 2254.29 | 2829.85 | 0.327922 | 0.13237 |
| Cluster-4169.19122 | Mitogen-activated protein kinase kinase kinase 5 | 86.66666667 | 75.66333 | -0.19349 | 0.243613 |
| Cluster-6520.0 | Mitogen-activated protein kinase kinase kinase 5 | 3.333333333 | 6 | 0.691878 | 0.53843 |
| Cluster-4169.5953 | Mitogen-activated protein kinase kinase kinase 20 | 675.6666667 | 2018 | 1.577124 | 0.000427 |
| Cluster-4169.6142 | Mitogen-activated protein kinase kinase kinase 17 | 1356.316667 | 1277.333 | -0.08649 | 0.876878 |
| Cluster-4169.9006 | phosphatidylinositol phospholipase C | 2228.58 | 2982.97 | 0.420461 | 0.019016 |
| Cluster-4169.4260 | phospholipase C | 46.33333333 | 341.3333 | 2.854473 | 0.05088 |
| Cluster-4169.7749 | phospholipase C | 4628.43 | 9741.027 | 1.073387 | 0.001034 |
| Cluster-4169.7339 | phospholipase C | 44.33333333 | 321.3333 | 2.829909 | 0.159505 |
| Cluster-4169.5311 | phosphatidylinositol phospholipase C activity | 1744.03 | 1536.697 | -0.18248 | 0.035082 |
| Cluster-4169.13635 | phospholipase C | 304.6666667 | 342.3167 | 0.167581 | 0.187285 |
| Cluster-4169.15331 | phosphatidylinositol phospholipase C | 90.33333333 | 70.00333 | -0.36325 | 0.196396 |
| Cluster-4169.15330 | phosphatidylinositol phospholipase C | 95.66666667 | 79.33333 | -0.26702 | 0.377148 |
| Cluster-4169.12716 | protein kinase C substrate 80K-H | 862 | 1013 | 0.232625 | 0.282035 |
|  |  |  |  |  |  |
| Cluster-647.0 | Protein kinase 2 | 0 | 0 | 0 | 0 |
| Cluster-4169.5809 | dopamine binding | 687.3333333 | 981.6633 | 0.51359 | 0.068014 |
| Cluster-4169.10520 | calmodulin binding | 7055.936667 | 6860.267 | -0.04057 | 0.858216 |
| Cluster-4169.1218 | calmodulin binding | 7 | 2.666667 | -1.12553 | 0.241872 |
| Cluster-4169.17321 | calmodulin binding | 134.3333333 | 138.6667 | 0.045471 | 0.745253 |
| Cluster-4169.8222 | calmodulin binding | 4128.693333 | 6996.43 | 0.76079 | 0.025599 |
| Cluster-4169.12861 | calmodulin binding | 988 | 884.92 | -0.15879 | 0.119259 |
| Cluster-4169.20109 | calmodulin binding | 12 | 2 | -2.11548 | 0.012088 |
| Cluster-4169.2194 | calmodulin binding | 106.6666667 | 66 | -0.68434 | 0.036263 |
| Cluster-4169.1452 | calmodulin binding | 12.33333333 | 21.33333 | 0.744161 | 0.02067 |
| Cluster-4169.10279 | calmodulin binding | 735.7766667 | 210.1133 | -1.80321 | 0.001792 |
| Cluster-4169.8326 | calmodulin binding | 1721.286667 | 4198.51 | 1.285896 | 0.001513 |
| Cluster-4169.7910 | calmodulin binding | 1945.666667 | 8877.1 | 2.189245 | 0.016563 |
| Cluster-4169.5384 | phosphatidylinositol-4,5-bisphosphate 4-phosphatase activity | 1823.333333 | 899.9933 | -1.01778 | 0.005421 |
| Cluster-4169.11161 | phosphatidylinositol-4,5-bisphosphate 4-phosphatase activity | 312 | 379.3333 | 0.281102 | 0.040173 |
| Cluster-4169.7620 | phosphatidylinositol-4,5-bisphosphate 4-phosphatase activity | 960.6666667 | 2147.333 | 1.159609 | 0.000283 |
