# Supplementary material for: Cadmium stress triggers dopamine signaling in duckweed (Lemna turionifera 5511) revealed by a fluorescent biosensor
Source: Front Plant Sci. 2026 Jun 1;17:1833157. doi: 10.3389/fpls.2026.1833157 (PMC13265539; doi:10.3389/fpls.2026.1833157)
Supplement: Supplementary file 2 [file DataSheet1.docx]

| 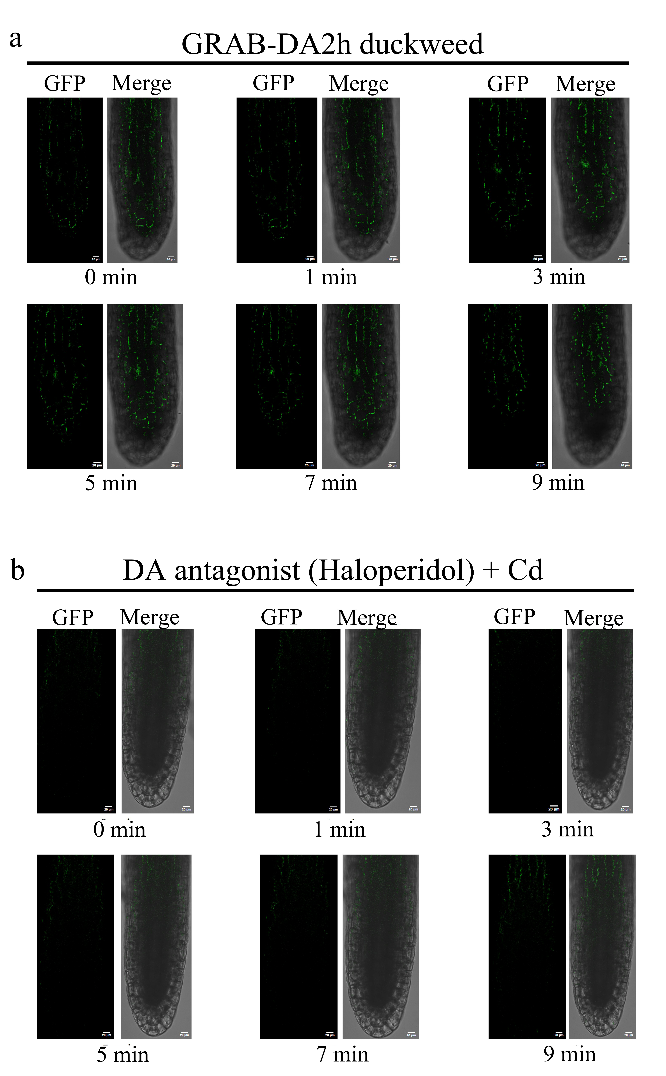Figure S1. Effects of haloperidol pretreatment on the dynamic changes of dopamine signals in duckweed roots under cadmium stress. (a) Dynamic changes of GFP fluorescence signals in the roots of duckweed expressing the GRAB-DA2h dopamine sensor at 0–9 min under 1.96×10⁻³ M CdCl₂ stress. (b) Dynamic changes of GFP fluorescence signals in the roots of GRAB-DA2h-expressing duckweed pretreated with 1 mg/L haloperidol for 2 h, under the same cadmium stress conditions over 0–9 min. All images were acquired by laser scanning confocal microscopy. Scale bars: 20 μm. |
| --- |
